# Sudden Death Due to Coronary Vasculitis: An Unexpected Autopsy Finding and Unusual Association With Thymic Hyperplasia

**DOI:** 10.7759/cureus.51531

**Published:** 2024-01-02

**Authors:** Aryel Brookins, David M Waters, Lorenzo Gitto

**Affiliations:** 1 Pathology, Cook County Medical Examiner's Office, Chicago, USA

**Keywords:** inflammation, autoimmune disorders, thymic hyperplasia, coronary vasculitis, autopsy

## Abstract

A 43-year-old Asian woman was found unresponsive on the sidewalk, prompting a call to 911. Upon transportation to the hospital, no signs of trauma were evident, but her condition deteriorated, leading to her eventual passing. An autopsy revealed a complete occlusion of the right coronary artery due to segmental coronary artery vasculitis. Without details of the individual's social or medical history, the specific type of coronary vasculitis couldn't be determined. The internal examination also showed thymic hyperplasia of unknown origin. While there is no conclusive evidence, a potential link between thymic hyperplasia, immune dysregulation, and coronary artery vasculitis is discussed, considering the case and existing literature.

## Introduction

Coronary artery vasculitis (CAV) is a broad term referring to the swelling or inflammation of the walls of a coronary artery, potentially leading to aneurysm, occlusion, rupture, or stenosis. This condition can arise from autoimmune disorders, infections, or drug reactions. The epidemiology of coronary vasculitis varies based on the subtype of vasculitis. Therefore, deriving overall epidemiological data is challenging. Still, more information is available for the most common types of vasculitis affecting coronary arteries, including polyarteritis nodosa, Kawasaki’s disease, Takayasu’s arteritis, giant cell arteritis, and more recently, severe acute respiratory syndrome coronavirus 2 (SARS-COV-2) [1]. Polyarteritis nodosa shows an incidence of 4-10 cases per million per year, predominantly affecting males over 40 years without racial predilection. Kawasaki disease presents an incidence of two cases per million per year, primarily impacting children under five years, with a predisposition toward Asian ancestry. Takayasu arteritis and giant cell arteritis demonstrate incidences of 1-3 cases per million per year. Takayasu arteritis predominantly affects females under 40 years, also showing a preference for Asian ancestry. In contrast, giant cell arteritis mostly affects females over 50 years, with a tendency toward White populations, particularly those of Scandinavian ancestry [2]. Currently, there is still limited epidemiological data on SARS-CoV-2-related coronary vasculitis, and further studies are needed to comprehend the actual prevalence of this phenomenon. CAV rarely results in sudden cardiac death, accounting for only about 12% of non-atherosclerotic sudden cardiac deaths [3].

Thymic hyperplasia is defined as the enlargement or overgrowth of the thymus gland and is associated with several conditions, mostly immunological disorders [4]. The incidence of occurrence of thymic hyperplasia is highest between the ages of 40 and 50 years old. It was also found to be more common in African-American and Asian populations compared to other races [5]. While no definitive link between CAV and thymic hyperplasia has been established, this report and a literature review identified two cases where both conditions coexisted. The discussion explores a potential association between CAV and thymic hyperplasia.

## Case presentation

A 43-year-old Asian woman was found unresponsive outdoors on the sidewalk by a passerby who alerted Emergency Services. Upon arrival, the subject was found to be apneic and pulseless, scoring 3 on the Glasgow Coma Scale. An electrocardiogram was performed, revealing asystole. Cardiopulmonary resuscitation was initiated in the ambulance, and she was transported to the local hospital. Unfortunately, her condition did not improve, and despite 30 minutes of continuous resuscitation efforts, she was pronounced dead in the Emergency Department. Due to the lack of medical history and circumstantial data, the body was referred to the Medical Examiner’s Office for a forensic autopsy.

**Figure 1:**
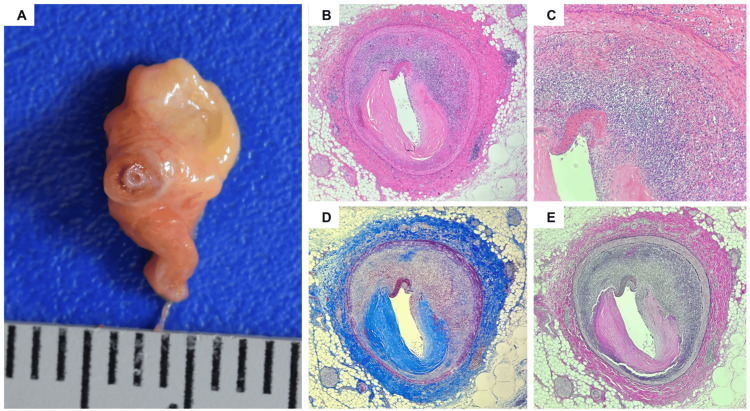
Right coronary artery (gross and microscopic examination) A. Gross finding showing critical lumen compression. B-C. Diffuse lymphocytic infiltrates involving predominantly the media, with areas of adventitial and intimal involvement, with multiple foci of disrupted elastic lamina, marked lumen stenosis, and intimal atherosclerotic plaque (B – H&E 40X; C – H&E 100X). D. Masson trichrome stain: patchy areas of fibrosis within the coronary artery wall. E. Verhoeff elastin: areas of disrupted adventitial elastic lamina.

An autopsy examination revealed no evidence of traumatic injuries. Examination of the heart revealed a pinpoint right coronary artery lumen without clear evidence of intraluminal thrombus or atherosclerotic deposits (Figure 1A). The left anterior descending and left circumflex coronary arteries showed non-critical stenosis.

The heart weighed 283 grams. The heart chambers and valves were grossly unremarkable. The right ventricle wall was 0.3 cm, the left ventricle wall was 1.0 cm, and the interventricular septum was 1.0 cm thick. The posterior wall of the left ventricle showed an ill-defined area of intraparenchymal hemorrhage extending from the subaortic plane to 1.5 cm above the apex. Additional relevant findings included an enlarged 50-gram, pink-gray thymus surrounded by an intact capsule. Sectioning of the organ revealed pale pink, homogeneous, and soft cut sections with a lobulated appearance and spots of yellow adipose tissue., with no gross lesions, masses, or other abnormalities.

**Figure 2:**
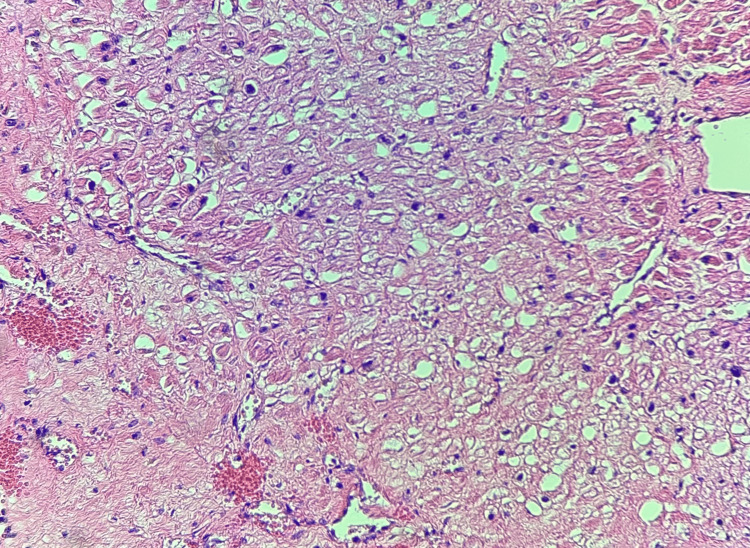
Heart histology Diffuse myocardial vacuolization (H&E, 100X)

Microscopic examination of the right coronary artery (Figures 1B-1E) demonstrated diffuse lymphocytic infiltrates in the arterial wall predominantly involving the media, with areas of adventitial and intimal involvement, with marked lumen stenosis and multiple foci of disrupted elastic lamina. Masson trichrome and elastin stains highlighted the disruption of the media and adventitial architectures and the disruption of the elastic lamina. Sections of the myocardium showed findings consistent with acute and subacute myocardial ischemia, including vacuolation of cardiomyocytes (Figure 2), foci of intraparenchymal hemorrhage and coagulative necrosis, granulation tissue, and lymphocytic infiltrates within the posterior wall of the left ventricle, interventricular septum, and right ventricle. Postmortem toxicology on peripheral blood was negative for alcohol, prescription drugs, or drugs of abuse.

The cause of death was certified as coronary artery vasculitis, and the manner of death was natural.

## Discussion

Diagnosing a specific subtype of vasculitis can be challenging due to their overlapping clinical and pathologic findings. Since CAV and its sequelae present a poor prognosis, prompt diagnosis and early institution of therapy can result in higher survival rates. The clinical presentation may lack specificity, but potential clinical indicators pointing toward an inflammatory origin include unexplained elevated inflammatory markers or signs of active inflammation. A mandatory step to precisely identify the specific type of vasculitis involves a histopathological examination. Nevertheless, it should be considered that different types of vasculitis may share similar histopathologic findings [6]. Thus, integrating clinical data allows for a more comprehensive assessment of the patient's medical history, presenting symptoms, and other relevant factors that might contribute to the development and manifestation of coronary vasculitis. Unfortunately, medical history and clinical presentation are often unknown during autopsy.

In the present case, the lack of information regarding the decedent’s social and medical history prevented the exact determination of the type of coronary artery vasculitis. However, the diffuse lymphocytic infiltrates seen in the coronary wall may suggest an immune-mediated process, potentially indicating an immune response to an underlying infection or autoimmune disease [7].

An interesting finding in this case was the presence of thymus hyperplasia (50 grams) discovered during autopsy. Thymic hyperplasia is the enlargement or overgrowth of the thymus gland caused by various factors such as infections, autoimmune disorders, and certain medications [4]. An enlarged thymus in an adult may represent thymic hyperplasia, which is generally characterized by an increase in the size of the thymus gland without an apparent cause, and it can be classified into two types: true thymic hyperplasia and lymphoid hyperplasia. True thymic hyperplasia is a diffuse enlargement associated with an increase in both thymic tissue and fat. In contrast, lymphoid hyperplasia is an enlargement due to increased germinal centers and lymphoid follicles, which may be associated with autoimmune disorders or inflammatory processes [8].

A review of the English literature yielded only a single case report of a sudden death due to coronary vasculitis associated with an enlarged thymus in a younger individual [9]. The case involved an 18-year-old male who experienced sudden fatigue while playing soccer, subsequently collapsing upon sitting on a bench. An autopsy examination revealed a heart weighing 320 grams with focal nodular areas on the anterior interventricular and circumflex coronary arteries, causing at least 80% luminal obstruction. Multiple myocardial scars were observed, indicating a recent infarction in the left ventricle. Additionally, both the spleen and thymus were enlarged. The authors suggest that, after excluding other potential causes based on the absence of specific criteria, the most probable diagnoses appeared to be either Kawasaki disease or polyarteritis nodosa. They do not comment about a possible association between CAV and thymic hyperplasia. The subject's race was not disclosed. Unfortunately, in our case, the lack of information regarding the circumstances of death prevents us from any comparisons with the case reported in the literature.

While establishing a direct link between CAV and thymic hyperplasia remains uncertain, autoimmune conditions associated with thymic hyperplasia could potentially influence the development and progression of CAV. Generally, autoimmune diseases show strong associations with polymorphisms in genes preferentially expressed in T cells [10], contributing to systemic autoimmunity across various conditions, including vasculitis [11]. Individuals with autoimmune disorders often exhibit decreased numbers and impaired function of regulatory T cells, likely contributing to the disruption of vascular immune homeostasis [12]. The heart frequently becomes a target of autoimmune conditions, leading to various complications, including microcirculatory issues [13].

## Conclusions

Coronary artery vasculitis should be included in the differential diagnosis of sudden deaths in young adults. Thymus persistence and hyperplasia in adults are generally associated with multiple autoimmune conditions since T-cells generated by an abnormal thymus may be impaired and exert local or systemic damage. An intricate relationship between thymic hyperplasia and coronary artery vasculitis warrants further investigation since a potential interplay role between these two conditions cannot be completely excluded.

## References

[1] Gori T (2021). Coronary vasculitis. Biomedicines.

[2] Khanna S, Garikapati K, Goh DS, Cho K, Lo P, Bhojaraja MV, Tarafdar S (2021). Coronary artery vasculitis: a review of current literature. BMC Cardiovasc Disord.

[3] Bukiri H, Ruhoy SM, Buckner JH (2020). Sudden cardiac death due to coronary artery vasculitis in a patient with relapsing polychondritis. Case Rep Rheumatol.

[4] Sun L, Li H, Luo H, Zhao Y (2014). Thymic epithelial cell development and its dysfunction in human diseases. Biomed Res Int.

[5] Rich AL (2020). Epidemiology of thymoma. J Thorac Dis.

[6] Baker-LePain JC, Stone DH, Mattis AN, Nakamura MC, Fye KH (2010). Clinical diagnosis of segmental arterial mediolysis: differentiation from vasculitis and other mimics. Arthritis Care Res (Hoboken).

[7] Kulshrestha V (2020). Coronary vasculitis - a case report. Int J Recent Sci Res.

[8] Mollaeian A, Haas C (2020). A tale of autoimmunity: thymoma, thymectomy, and systemic lupus erythematosus. Clin Rheumatol.

[9] Dermengiu D, Hostiuc S, Cristian Curca G, Constantin Rusu M, Paparau C, Ceausu M (2014). Sudden death due to isolated segmentary coronary vasculitis. Am J Forensic Med Pathol.

[10] Marson A, Housley WJ, Hafler DA (2015). Genetic basis of autoimmunity. J Clin Invest.

[11] Bluestone JA, Bour-Jordan H, Cheng M, Anderson M (2015). T cells in the control of organ-specific autoimmunity. J Clin Invest.

[12] Jin K, Parreau S, Warrington KJ, Koster MJ, Berry GJ, Goronzy JJ, Weyand CM (2022). Regulatory T cells in autoimmune vasculitis. Front Immunol.

[13] Pan SY, Tian HM, Zhu Y (2022). Cardiac damage in autoimmune diseases: target organ involvement that cannot be ignored. Front Immunol.

